# Effect of Volatile Organic Compounds from Branches of Healthy and Unhealthy *Araucaria araucana* (Molina) K. Koch Trees on Host Selection by Bark Beetle *Sinophloeus porteri* (Coleoptera: Curculionidae)

**DOI:** 10.3390/insects16070712

**Published:** 2025-07-10

**Authors:** Washington Aniñir, Leonardo Bardehle, Cristian Montalva, Andrés Quiroz, Javier Espinoza

**Affiliations:** 1 Doctorado en Ciencias de Recursos Naturales, Universidad de La Frontera, Av. Francisco Salazar 01145, Casilla 54-D, Temuco 4811230, Chile; w.aninir01@ufromail.cl; 2 Laboratorio de Química Ecológica, Departamento de Ciencias Químicas y Recursos Naturales, Universidad de La Frontera, Av. Francisco Salazar 01145, Casilla 54-D, Temuco 4811230, Chile; javier.espinoza@ufrontera.cl; 3 Centro de Investigación Biotecnológica Aplicada al Medio Ambiente (CIBAMA), Universidad de La Frontera, Av. Francisco Salazar 01145, Casilla 54-D, Temuco 4811230, Chile; 4 Departamento de Producción Agropecuaria, Universidad de La Frontera, Av. Francisco Salazar 01145, Casilla 54-D, Temuco 4811230, Chile; leonardo.bardehle@ufrontera.cl; 5 Laboratorio de Salud de Bosques, Instituto de Conservación, Biodiversidad y Territorio, Facultad de Ciencias Forestales y Recursos Naturales, Universidad Austral de Chile, Valdivia 5090000, Chile; cristian.montalva@uach.cl

**Keywords:** *Araucaria araucana*, *Sinophloeus porteri*, volatile organic compounds (VOCs), olfactometry

## Abstract

*Araucaria araucana* is one of the longest-living Chilean trees. Recently, Araucaria Leaf Damage disease, which causes damage to branches and crowns, was detected. *Sinophloeus porteri*, a bark beetle affecting *A. araucana*, could be associated with foliar damage. However, little is known about their ecological and chemical interactions. This study examined the olfactory response of *S. porteri* to volatiles emitted from *A. araucana*. Branches and weevils were collected from a national park, and volatiles were trapped from both healthy and unhealthy branches. Thirty terpenes were identified, some of which were reported for the first time in *A. araucana*. Healthy branches emitted large amounts of myrcene (>360 ng g^−1^ day^−1^), and unhealthy branches showed high hibaene emanations (>140 ng g^−1^ day^−1^). Olfactory assays verified that *S. porteri* was attracted to the volatile blends of branches, regardless of the health condition of the branches, but preferred the blend of unhealthy branches. Moreover, myrcene was repellent to these weevils, and hibaene acted as an attractant, suggesting that *A. araucana* might use myrcene for defense against *S. porteri*, and hibaene could stimulate host selection by beetles.

## Simple Summary

*Araucaria araucana* is one of the oldest trees in Chile, with a lifespan of up to 1000 years. Recently, a disease known as Araucaria Leaf Damage (ALD) has been affecting these trees, causing damage to their branches and crowns. Bark beetles, including *Sinophleous porteri*, may contribute to this problem, either by directly damaging the trees or by spreading harmful fungi. However, little is known about how these insects interact with these trees. This study examines whether volatile organic compounds released by the trees affect the behavior of *S. porteri*. We collected branches and beetles from a national park and analyzed the chemical compounds released by healthy and unhealthy trees. Myrcene, the most abundant compound emanated by healthy branches, was repellent to this weevil. Hibaene, found in high amounts in unhealthy branches, was attractive to *S. porteri* beetles. These findings help us understand how beetles respond to tree cues and may inform future strategies for monitoring and protecting these ancient trees. Understanding these interactions is crucial to conserving *A. araucana*, especially as environmental changes threaten its survival.

## 1. Introduction

*Araucaria araucana* (Molina) K. Koch, also known as araucaria or pehuén among the native people, is a conifer endemic to Chile and Argentina. In Chile, it is distributed from Nahuelbuta Mountain (between 37°20′ and 38°40′ S) to the Andes Mountains (between 37°30′ and 39°30′ S) [1,2]. On the Argentinean side of the Andes Mountains, *A. araucana* is distributed in the Ruca Choroy and Pulmari areas, with a significant portion of the population residing in the Lanín National Park (40°03′ S; Neuquén Province) [1]. It is categorized as one of the oldest Chilean trees in its flora, having lived for approximately 1000 years. This ancestral tree produces a fruit-like pine kernel called Pehuén, which serves as a food source for the local ethnic group, the Pehuenches (people of the Pehuén) [3,4]. For this reason, among others, *A. araucana* was declared a national monument and a vulnerable species in 1990 by Decree No. 43 of the Ministry of Agriculture of Chile, which prohibited its exploitation.

*A. araucana* has suffered a severe decline in the last few years. To date, the causes of this phenomenon remain unclear. Thus, diverse factors that could be potentially involved in the disease process have been considered, including abiotic factors like, winds, dryness, snow, floods, and climate change in general [5,6] and biotic factors like nematodes, insects, bacteria, and fungi, among others [7,8,9]. In this context, several fungi have been isolated from the branches, including *Diaporthe araucanorum*, which can exhibit endophytic, saprotrophic, and phytopathogenic characteristics [10]. *Mortierella* species have been isolated from necrotic tissue of the phloem and xylem, where two *Mortierella* spp. showed pathogenicity [11]. The presence of a new species, *Ophiostoma pehueninum*, was established by Zapata et al. [12], which differs from the previously reported *O. araucariae* [13]. *A. araucana* in Chile was shown to cause canker-like symptoms similar to those observed under natural conditions. This could implicate them as the cause of the damage [14]. However, the effects of fungi on trees have been widely reported [11,12,15,16]. It has been described that curculionids play a crucial role in causing foliar damage and dieback in conifers, which in some cases can result in the death of the entire tree [17,18], either through direct damage or through the transmission of microorganisms. Such is the case with the genus *Dendroctonus*, one of the largest pest insects of pine, including *Dendroctonus frontalis* and *Dendroctonus terebrans*, which are among the most significant, along with those of the genus *Ips* [19,20].

*A. araucana* presents one of the richest faunas of Curculionidae. It includes 23 species, of which 12 are associated with the strobilus, 11 with the bark, and 2 with the xylem [21]. Several beetles have been associated with dead branches of *A. araucana*, including the weevils *Araucarios major*, *Araucarios minor*, *Xylechinosomus valdivianus* (Eggers) (=*Xylechinosomus bicolor*), and *Sinophleous porteri* Briggs (Coleoptera: Curculionidae) (=*Sinophloeus destructor* (Eggers)) [21]. Considering that these species are part of the *A. araucana* ecosystem and have been linked to damage as secondary agents, little is known about the aggressiveness they can exhibit during periods of climatic disturbance [22]. It has been reported that insects undergoing temperature changes can transition from an endemic life stage (most commonly observed in *A. araucana*) to an epidemic state [23]. These insects that manage to reach the epidemic stage can bypass the defenses of healthy trees, unlike those in the endemic stage, which require the tree to be under some stress or have its optimal condition diminished [24].

On the other hand, volatile organic compounds (VOCs) emitted from plants, including higher plants, can act as signals in chemical communication with insects, including endemic and epidemic species [25,26]. Most of the VOCs emanated from higher plants are terpenoids [27,28], which are not different from those of *A. araucana*. The essential oil from leaves and branches and the oleoresin of *A. araucana* mainly constitute terpenes [29,30,31,32,33,34,35,36]. Terpenes play important biological roles in forest ecosystems [37,38], such as attracting pollinators or herbivores [39,40,41], defending against bark beetles [42,43], and even playing a role in the enzymatic machine of a forest with the capacity to decrease specific enzymatic activity like abietic acid over *β*-glucosidase [44]. Moreover, a significant increase in terpenoid emissions, produced by a massive bark beetle attack, can alter foliage flammability based on their high heat values, low flash points, and low flammability limits [45,46]; therefore, in the event of a massive bark beetle infestation, there would be a greater chance of forest fires, justifying bark beetle supervision.

Since there is limited information on the role of terpenes in host tree colonization by curculionids, and *S. porteri* has been found in its largest population in Chilean *A. araucaria*, this study investigated the effect of terpenes released from *A. araucana* on the olfactory responses of *S. porteri* adults. The identification of the terpenes responsible for the attraction elicited from *A. araucana* by *S. porteri*, is a key step in understanding the establishment of curculionids on plants and in developing a monitoring program based on attractant terpenes.

## 2. Materials and Methods

### 2.1. Plant Material Collection

Branches (including leaves) of *A. araucana* were collected from the Lagunas sector in the Conguillio National Park (PNC), La Araucania, Chile (38.6350290° S, 71.6488440° W), between February and May 2021. PNC is the largest national park in the range of distribution of *Araucaria araucana*. The size of the plots was established at 500 m^2^, as stipulated by Mueller-Dombois and Ellenberg [47], and the distribution was established randomly. The collection consisted of selecting three trees with evidence of foliar damage and another three trees without evidence of foliar damage. Three branches, including leaves, were taken from each tree, totaling nine branches per tree. Branches were labeled and transported inside a container at 0 °C ± 4 °C to the Laboratorio de Química Ecológica at Universidad de La Frontera (Temuco, Chile), where branches were kept individually at −18 °C ± 4 °C in an inert paper bag until analysis. Plant material and insect collection was approved by Corporación Nacional Forestal de Chile (Authorization No. 009/2019).

### 2.2. Insect Collection

The insects for olfactometry were collected from branches obtained in Conguillio National Park, La Araucanía region, southern Chile, between December 2020 and April 2021. Three 30 cm long branches were selected from two trees in each plot for active collection. These branches were extracted with a 15 m high telescopic pole with a branch cutter at the end, which allows access to branches up to 16 m high. Branches were maintained in the laboratory at 16 °C ± 1 °C in darkness until the olfactometric bioassays were conducted. To select the samples from which the insects were collected, the branches had been previously observed for signs of insect presence, as indicated by chlorosis and brown leaf discoloration. The insects were removed from the branches 24 h before the bioassay and maintained in Petri dishes without food at 20 °C ± 1 °C in darkness. Before the assays, the insects were observed, and those that were walking and active were selected for olfactory experiments.

### 2.3. Volatile Capture from Branches and Leaves of A. araucana

Volatiles from the branches and leaves of *A. araucana* were absorbed into a Porapak Q cartridge using a positive/negative-pressure air system, according to the methodology proposed by Mueller-Dombos et al. [47] and Barrios-San Martin et al. [48]. Thirty cm of each branch was enclosed in a 1325 mL Pyrex glass chamber. Purified air was pumped into the bag at a flow rate of 1 L/min under light exposure, and another pump drained purified air at the same rate toward a cartridge containing Porapak Q (100 mg; 80 Å, 100 mesh), where the terpenes released by *A. araucana* were adsorbed for 24 h. The trapped volatiles were desorbed from the Porapak Q filter using hexane (GC grade; Merck, Darmstadt, Germany) (2 mL) and maintained at −4 °C until chemical analysis or bioassay development [49,50].

### 2.4. Analysis of Volatile Compounds by GC-MS

The presence of terpenes was analyzed by GC-MS using a Thermo Electron Model Trace 1300 (Thermo Fisher Scientific Inc., Waltham, MA, USA) coupled to an ISQ 7000 Thermo Electron quadrupole mass spectrometer with an integrated data system (Xcalibur 4.1, Thermo Fisher Scientific Inc., Waltham, MA, USA). Separation was carried out by a BP-5 capillary column of 30 m length (0.25 μm film thickness × 0.25 mm i.d., SGE Forte, Trajan Scientific and Medical, Ringwood, VIC, Australia) with helium gas as a carrier (1.0 mL/min) at an initial temperature of 40 °C for 2 min, which was increased to 250 °C with a 5 °C increment/min, held for 5 min. Both the injector and interface temperatures were maintained at 250 °C, while the detector temperature was set at 250 °C. The mass detector employed an ionization energy of 70 eV. Recording conditions employed a scan time of 1.5 s and a mass range of 30 to 400 amu. The compounds were identified by a comparison of their mass spectra with those in the NIST version 2.0 library database (NIST, Gaithersburg, MD, USA), by a comparison of their calculated retention index with those reported in the literature for the same type of stationary phase, and, in some cases, by the co-injection of commercial standards. Calculated retention indices were determined through the retention time of C9–26 n-alkane standards (100 μg/mL in hexane) (Sigma-Aldrich, St. Louis, MO, USA) using the equation described by Kovats and Keulemans [51]. Moreover, GC peaks will be coincidental and compounds identical if their retention times do not differ by more than ± 0.03 min and the similarity index of their mass spectra [52] is greater than 95%.

Equation (1) was used to calculate the concentrations of the compound released by each sample, where *A* is the area of the compound in the chromatogram; *I* represents the intercept in the equation of the line; *S* is the Slope; and *Wg* is the weight of the branches in grams. Five concentrations of α-pinene were used for obtaining the calibration curve.

(1) $$\left[Emanated\;compound\right]ng/g=\left(\left(2\left(\frac{A*I}{S}\right)\right)/1000*Wg\right)*1000$$

### 2.5. Hibaene Isolation

*A. araucana* leaves (151.43 g) were ground and macerated in 1000 mL of dichloromethane (DCM) for 5 days at room temperature [53]. The resultant suspension was filtered through filter paper, and the water present in the extract was trapped with anhydrous Na_2_SO_4_. The solution was filtered, and the DCM was evaporated under reduced pressure, yielding a DCM extract. The DCM extract was fractionated by column chromatography using silica gel as the stationary phase. The elution was performed using n-hexane/DCM (1:1), and the resultant fractions were analyzed by thin-layer chromatography on silica gel 60 F254 pre-coated plates, using a *p*-anisaldehyde–sulfuric acid spray reagent for detection [54]. Similar fractions were grouped (8.94 mg) and then analyzed by GC-MS (hibaene-rich solutions: 70% of hibaene). Afterward, this solution was suspended in 5 mL of ethyl acetate. The suspension was heated to solubilize the diterpene, and the hot solution was filtered under reduced pressure. The white crystalline precipitate obtained was filtered and dried. A solution of 10 ppm was prepared and analyzed by GC-MS under the previously described conditions. The purity of the hibaene obtained was 97%, and it was used as a reference standard.

### 2.6. Olfactometry

Bioassays were performed in a four-arm olfactometer (height, 6 mm; narrowest width across, 35 mm; inside diameter of air inlet tubes, 5 mm; diameter across, 100 mm), used to assess the olfactory response of *S. porteri* according to Parra et al. [49] with modifications; the observation arena was divided into four arm zones, with an indifferent zone in the center, designated the zone of decision. Volatile blends from branches and leaves of *A. araucana* and pure compounds identified in these blends were tested. The pure compounds used here were purchased from Sigma-Aldrich (St. Louis, Mo, USA), including α-pinene (≥97.5% purity (determined by GC); CAS N°: 80-56-8; product number: 147524), L-limonene (96% purity (determined by GC); CAS N°: 5989-54-8 product number: 218367), camphor (96% purity (determined by GC); CAS N°: 76-22-2; product number: 148075), and myrcene (90% purity (determined by GC); CAS N°123-35-3; product number W276212). The volatile blend tested here was obtained by trapping volatiles with Porapak Q, as described in Section 2.3. Isolated hibaene was obtained according to Section 2.5 and tested in this study.

Strips of filter paper (0.5 cm × 5.0 cm) were individually loaded with 10 µL of solutions of pure compounds of the following samples: (a) camphor, myrcene, α-pinene, and limonene at 20, 25, 50, and 100 ppm, respectively; (b) volatile blends from healthy and unhealthy branches; (c) isolated hibaene at 10 ppm. The concentration values were obtained from the literature for curculionids on *Pinus* species that responded to electroantennographic stimuli. For blanks, filter papers were loaded with 10 µL of *n*-hexane. After the compounds were loaded, the filter paper was exposed to air for 20 s to allow solvent evaporation and then inserted into a glass Pasteur pipette. Two lines of air coming from the stimulus treatment were connected in the opposite corners of the arena; the other two lines connected to the blank. The olfactometer was horizontally positioned over a white LED flat panel light, with a room temperature of 21 °C ± 1 °C and a humidity level of 30–50%. The center of the olfactometer was connected to a vacuum pump generating a purified air flow (200 mL min^−1^) that converged with the stimuli in the center. The air was purified through activated carbon.

The *S. porteri* responses to the samples were tested during the photophase (from 9 h to 14 h) under a 12:12 h light/dark photoperiod. Unsexed *S. porteri* weevils of unknown age and 24 h starved weevils were tested individually. The insect was released into the center of the olfactometer through a 5 mm hole. The time spent in the different areas of the observation arena by each individual was recorded for 30 min (each insect was considered as a repetition, and insects were used only once). Thirty insects were tested for each compound, and the olfactometer was rotated to a new position for each repetition. The behavior of the insect in the arena was recorded by the Ethovision 3.1.16 program (Noldus Information Technology, Wageningen, The Netherlands). The total time spent in the stimulus arms was compared with the time spent in the control arms.

### 2.7. Statistical Analysis

One-way analysis of variance (ANOVA; *p* ≤ 0.05) was performed to compare the time spent by *S. porteri* in each olfactometer arm. Multiple comparisons were performed by applying the Tukey test. Principal component analysis (PCA) was performed using JMP 16 software (SAS Institute Inc., Cary, NC, USA, 1989–2023). PCA was used to determine the sample cluster as a function of the combination of variables.

## 3. Results

Volatile blends from healthy and unhealthy *A. araucana* branches were analyzed by GC-MS (Table 1). A total of 25 compounds were identified in each blend, corresponding to 99.90% of detected compounds from healthy branches and 98.29% of detected compounds from unhealthy branches. In the volatile blend from healthy branches, eight monoterpenes (45.0%)— seven hydrocarbons (42.9%) and one oxygenated (2.1%)—12 sesquiterpene hydrocarbons (32.8%), and four diterpenes (22.2%)—three hydrocarbons (19.5%) and one oxygenated (2.7%)—were identified. In the volatile blend from unhealthy branches, five monoterpenes (18.6%)—four hydrocarbons (10.9%) and one oxygenated (7.7%)—14 sesquiterpenes (41.6%)—13 hydrocarbons (41.0%) and 1 oxygenated (0.7%)—and six diterpenes (39.7%)—four hydrocarbons (33.1%) and two oxygenated (6.7%)—were present.

**Figure 1:**
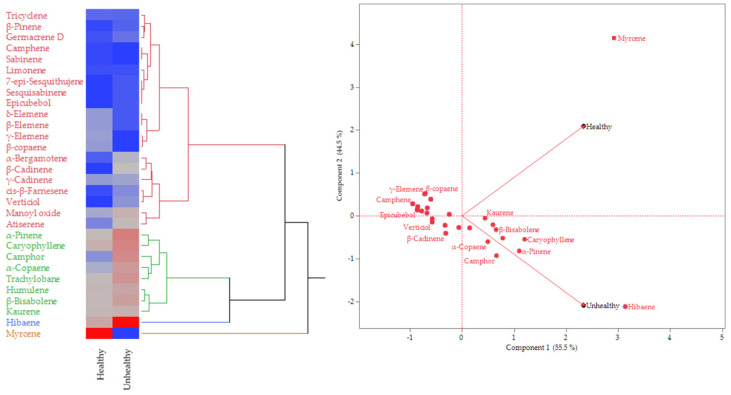
(**Left**) Heat map, including a dendrogram, showing the relative abundance of the 30 compounds present in volatiles from branches of healthy and unhealthy trees, from blue color (low abundance) to red color (high abundance). (**Right**) Exploratory principal component analysis (PCA): score plot and loading plot of the first two principal components for 30 terpenes from healthy and unhealthy branches.

The heat map, presented in Figure 1 (left), organizes the compounds from Table 1 by their concentration levels emitted from branches. The color blue indicates compounds present in low concentrations, while the color red signifies those present in high concentrations. According to the heat map and Table 1, myrcene (36.0%), a monoterpene hydrocarbon, was the most abundant compound in the healthy-branch blend, followed far behind by hibaene (8.1%, diterpene hydrocarbon) and caryophyllene (6.4%, sesquiterpene hydrocarbon). On the other hand, hibaene (17.6%) was the major component in the unhealthy-branch blend, followed by *α*-pinene (8.6%, monoterpene hydrocarbon), caryophyllene (8.1%), and camphor (7.7%, oxygenated monoterpene).

The dendrogram adjacent to the heat map (Figure 1) categorizes the compounds based on concentration variability among blends into four clusters, using color-coded lines. Myrcene showed the most significant abundance variation between healthy- and unhealthy-branch blends (brown), and hibaene presented a significant variation (blue). Compounds marked with green and red colors showed slight variations, with the latter exhibiting the lowest variation. Additionally, according to the score plot and loading plot in the principal component analysis (PCA) (Figure 1, right), myrcene and hibaene exhibit greater variability in terms of component 1, and hibaene shows the most significant variability in relation to component 2. Moreover, myrcene was the compound that most affected the composition of the volatile blend from healthy branches, and hibaene heavily influenced the volatile blend composition of unhealthy branches.

### Olfactometric Assays

**Figure 2:**
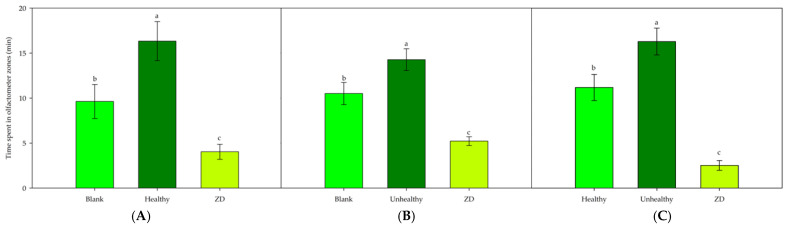
Olfactory response of *S. porteri* beetles exposed to volatile blends of *A. araucana* from (**A**) healthy branches, (**B**) unhealthy branches, and (**C**) healthy and unhealthy branches. ZD: zone of decision. The lines on the bars indicate the standard error. Different letters indicate significant differences based on one-way ANOVA and the HSD Tukey test (*p* ≤ 0.05), n = 30.

When *S. porteri* was exposed to the volatile blend from healthy branches, the average time spent by individuals in the treatment arms was significantly higher (16.33 min ± 2.17) than in the blank arms (9.63 min ± 1.89), and both were significantly higher than the time spent in the decision zone (4.03 min ± 0.83) (*p* < 0.05) (Figure 2A). Similarly, when *S. porteri* was exposed to volatiles from unhealthy branches, the average time spent by individuals in the treatment arms was significantly higher (14.28 min ± 1.24) than in the blank arms (10.51 min ± 1.21), and both were significantly higher than the time spent in the decision zone (5.21 min ± 0.49) (*p* < 0.05) (Figure 2B). However, when *S. porteri* was exposed to blends from both healthy and unhealthy branches, the average time spent by individuals in the arms with volatiles from unhealthy branches (16.29 min ± 1.49) was significantly higher (*p* < 0.05) than both in the arms with volatiles from healthy branches (11.18 min ± 1.45) and in the decision zone (2.52 min ± 0.54 min) (Figure 2C).

**Figure 3:**
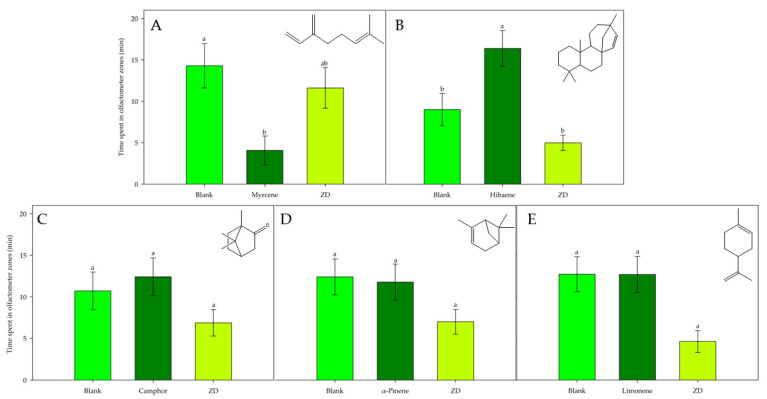
Olfactory response of *S. porteri* beetles exposed to single compounds identified in the volatile blends of branches, diluted in *n*-hexane. (**A**) Myrcene, (**B**) hibaene, (**C**) camphor, (**D**) *α*-pinene, and (**E**) limonene. Blank: hexane; ZD: zone of decision. The lines on the bars indicate the standard error. Different letters indicate significant differences based on one-way ANOVA and the HSD Tukey test (*p* ≤ 0.05), n = 30.

The influence exerted by myrcene and hibaene in the composition of volatiles from healthy and unhealthy branches, respectively, as indicated by PCA (Figure 1, left), suggests that these terpenes may have some noteworthy effects on *S. porteri*. Then, the olfactory response of *S. porteri* to commercial myrcene and isolated hibaene was evaluated and is presented in Figure 3. When the weevils were exposed to myrcene (diluted in *n*-hexane at 25 ppm), they showed a notable repulsion for this compound, spending an average time of 14.28 min ± 2.67 in the blank arms and 11.61 min ± 2.46 in the decision zone, and both average times were higher than the time spent in the treatment arms (4.06 min ± 1.74) (*p* < 0.05) (Figure 3A). On the contrary, hibaene was significantly preferred by the weevils (*p* < 0.05), where *S. porteri* spent 16.39 ± 2.16 min in the treatment arms, compared to 9.01 ± 1.90 min in the blank arms and only 4.94 ± 0.93 min in the decision zone (Figure 3B).

Although camphor, *α*-pinene, and limonene were grouped into the small and lowest-abundance-variation clusters (Figure 1, left), the olfactory response of *S. porteri* exposed to these monoterpenes was also evaluated. In tests with camphor, *α*-pinene, and limonene, there were no significant differences in the time spent by weevils between the blank arms and the treatment arms (Figure 3C–E).

## 4. Discussion

The volatile compounds emitted by healthy and unhealthy branches of *A. araucana* were collected and analyzed by GC-MS, and their compositions are reported in the present study for the first time. All detected compounds in these volatile blends were terpenes, distributed among monoterpenes (45.0% in HB and 18.6% in UB), sesquiterpenes (32.8% in HB and 41.6% in UB), and diterpenes (22.2% in HB and 39.7% in UB), including myrcene, hibaene, *α*-pinene, caryophyllene, and camphor, among others. Briggs and White [30] determined that essential oil (EO) from *A. araucana* leaves constituted ~25% monoterpenes, ~25% sesquiterpenes, and ~50% diterpenes, a similar percentage of terpenes to that found in this study. Similarly, Pietsh and König [34] obtained EO from *A. araucaria* leaves but using *n*-hexane as an extraction solvent, which was composed of sesquiterpenes at ~30% and diterpenes at ~70% [34]. The lack of monoterpenes in this EO is most likely due to the *n*-hexane evaporation process. In summary, previous studies showed that essential oils from *A. araucana* leaves contained terpenes, including geraniolene, limonene, *δ*-cadinene, (+)-cadinene, (−)-*α*-cadinol, *β*-caryophyllene, (−)-16-kaurene, (+)-15-beyerene, (−)-trachylobane, (−)-16-atisirene, 15-kaurene, isokaurene, atisirene, isoatisirene, (−)-rosa-5,15-diene, *ent*-13-*epi*-manoyl oxide, and (−)-sclarene [30,31,32,33,34]. There is no more information in the literature about the VOCs emitted from branches of *A. araucana* trees, though various compounds, as mentioned above, have been reported in the leaves of other species of the *Araucaria* genus, such as *Araucaria angustifolia*, *Araucaria bidwilli*, *Araucaria columnaris*, *Araucaria cunninghamii*, and *Araucaria heterophylla* [55,56]. Other chemical studies have been focused on the isolation and identification of secondary metabolites associated with medicinal properties attributed to the *A. araucana* resin and bark. Eleven diterpenes were isolated from the resin of Chilean *A. araucana*, which included ten labdane diterpenes and one pimarane [35]. Another study reported the secondary metabolite composition from the methanol extract of the heartwood of *A. araucana*. This extract contained secoisolariciresinol, eudesmin, lariciresinol, pinoresinol, and methoxy-pinoresinol, as well as mono-, di-, and triterpenes [36]. None of these compounds which are found in other plant organs were detected in the volatile blends released from healthy and unhealthy *A. araucana* branches, studied here.

Unlike previous studies, the present research focused on the volatile organic compounds of *A. araucana* branches that may be associated with foliar damage. In this context, the exploratory PCA revealed a clear separation between groups of healthy and unhealthy trees, as well as their VOCs. Furthermore, the analysis demonstrated the high influence of myrcene and hibaene in the volatile profiles of healthy and unhealthy trees. Consistently, myrcene was primarily released by healthy branches, and hibaene was the most emitted compound in unhealthy branches. The olfactory test with *S. porteri* adults revealed that this weevil was significantly attracted to volatile blends of healthy and unhealthy *A. araucana* branches, despite the differences among volatile profiles. The test indicated that the volatile compounds emitted by branches of *A. araucana* may be responsible, in part, for host selection by *S. porteri* adults. However, when *S. porteri* had the option to choose between volatiles from healthy and unhealthy branches, this weevil significantly preferred the volatile blend from unhealthy branches of *A. araucaria*, suggesting that the state of the branches affects the selection of *S. porteri*. Interestingly, the primary compound emanating from healthy branches was the monoterpene myrcene, whereas unhealthy branches did not release it. On the contrary, hibaene was the most released compound from unhealthy branches, and it was emitted to a lesser extent by healthy branches. This suggests that myrcene and hibaene may be associated with the host selection of *S. porteri*. Then, the olfactory response of this weevil to myrcene and hibaene was evaluated and reported in the present study for the first time. Myrcene and hibaene affected the behavior of *S. porteri* adults in different ways. Myrcene, the primary compound released by healthy branches, was repellent to the weevils. Hibaene, the most abundant compound in the unhealthy-branch blend, acted as an attractant. These results suggest that *A. araucaria* may utilize myrcene to prevent being selected by *S. porteri*. However, unhealthy trees are unshielded against *S. porteri* infestation. Moreover, an increased amount of hibaene released from these branches would allow their selection by *S. porteri*. There is no more information in the literature about the effects of VOCs emitted from *A. araucaria* trees on the host selection of *S. porteri*. However, the variation in terpene emissions by stressed conifers is not an isolated event. The amounts of several monoterpenes released from *Thuja occidentalis*, including myrcene, were increased in stressed trees infested with cypress bark beetle, *Phloeosinus aubei* [57]. Additionally, the use of terpenes as semiochemicals in choosing among host trees has been reported before for other curculionids. Successful host tree colonization and reproduction of *D. frontalis* and *D. terebrans*, two sympatric bark beetle pests that adversely affect *Pinus* spp., are dependent on a chemical communication system that includes compounds produced by both the beetles and their host trees [19]. Myrcene, *α*-pinene, *β*-pinene, and 4-allylanisole produced the strongest electroantennographic responses from *D. frontalis* and *D. terebrans.* Furthermore, field studies indicated that *α*-pinene, *β*-pinene, and 4-allylanisole significantly enhanced the attraction of *D. frontalis*, *D. terebrans,* and its major predator, *Thanasimus dubius,* to traps baited with attractive pheromones of both bark beetles. However, myrcene diminished this response for *D. frontalis*, acting as a repellent agent, similar to in the results of the present study [19].

In a similar way, the colonization of jack pine, *Pinus contorta*, by *Ips grandicollis* weevils was inhibited by the presence of lures baited with pheromones of mountain pine beetle (*Dendroctonus ponderosae*) and myrcene, a host volatile [58]. Another two pine bark beetles, *Tomicus piniperda* and *Hylurgops palliatus*, were capable of discriminating between different *Pinus* species through *β*-pinene emissions, the host-dominant volatile [59].

In the present study, hibaene showed a significant ability to attract *S. porteri* weevils, suggesting that this diterpene may be partially associated with the selection of *A. araucana* trees. Hibaene has been mainly found in the Araucariaceae family [33] and in some species of the Cupressaceae family, such as *Chamaecyparis obtusa* and *Chamaecyparis japonica* [53,60]. Hibaene has not been identified in VOCs from *Nothofagus pumilio, Nothofagus antarctica, Nothofagus dombeyi, Nothofagus alpina*, and *Nothofagus obliqua* [50], and furthermore, they are not infested by *S. porteri*. The lack of this diterpene may be the reason, in part, why trees of the *Nothofagus* genus are not suitable as hosts, despite being part of the same forest. Interestingly, Berasategui et al. [61] reported that some diterpenes can be digested by curculionids, thereby overcoming their toxicity and even enhancing the fecundity and hatching rate of the insects.

Camphor, *α*-pinene, and limonene were also selected for the olfactory tests, considering the well-known anti-insect properties of terpenes [62]. Although these compounds were minor components in the volatile blends of *A. araucaria* branches, they were not correlated with the differences among VOC profiles. Consequently, camphor, *α*-pinene, and limonene did not elicit a particular behavior of *S. porteri*. Similarly, no specific behavior was reported when the curculionids *Monochamus saltarius*, *Picea asperata*, *Pinus koraiensis*, and *Pinus tabuliformis* were exposed to camphor at concentrations of 10 µg µL^−1^ and 100 µg µL^−1^ [63]. Nevertheless, these results differed from the effects of these monoterpenes on other weevil species [19,64,65,66,67,68,69,70,71,72,73]. Faccoli et al. [64] and Munro et al. [19] reported that α-pinene was an attractant to the curculionids *Tomicus destruens*, *D. frontalis*, and *D. terebrans*. Furthermore, α-pinene proved helpful as a trap bait to catch *Ips sexdentatus* bark beetles under field conditions [65]. Males and females of *P. aubei*, and the bark beetles *Rhopalicus tutela* and *Rhopalicus mirus* showed an electroantennographic response when they were exposed to camphor at 10 µg µL^−1^ [66] and 30 ng µL^−1^ [67], respectively. Additionally, a blend containing camphor at 4.7 ng µL−1 elicited attraction in *R. tutela* and *R. mirus* [67]. Limonene has been reported to be part of the VOCs released by several species of conifers [68,69] and has elicited different effects on weevils associated with conifers [70]. Studying the toxicity of monoterpenes against the mountain pine beetle, *D. ponderosae*, Chiu et al. [70] mentioned that the two most toxic compounds, the two enantiomers of limonene, account for only 1–5% of the monoterpene profile of lodgepole pine, *P. contorta*, and an even lower percentage of the profiles of whitebark pine, *Pinus albiculus*, and ponderosa pine, *Pinus ponderosa*. Moreover, limonene can be used as an indicator of insect attack, as reported by Ghimire et al. [71], where the amount of limonene significantly increased when *Ips typographus weevils attacked Picea abies trees*. Related to the use of terpenes as trap baits, limonene acted synergistically with *α*-pinene to improve the catching of *Ips duplicatus* adults [72]. However, in the field, (*R*)-limonene significantly reduced the number of *Pityophthorus pubescens* beetles attracted to (+)-*trans*-pityol and racemic trans-pityol in traps in *Pinus radiata* (Pinaceae) stands [73].

The antecedents mentioned above are mainly based on volatile compounds from *Pinus* species and chemical communication with coleopteran guests, which calls us to pay special attention to the possible entry of weevils from nearby forest plantations, especially from *Pinus* spp., which are some of the principal invasive species in the habitat of *A. araucana* [74]. If they are present, some pest insects could detect *A. araucana* as a host and parasitize it. However, the effects of VOCs, including terpenes, on insect behavior rely mainly upon the stereochemistry and concentration of the semiochemicals, the beetle species, and the sex of the insects. Additionally, laboratory conditions do not precisely represent the natural conditions of wild *S. porteri* life. Then, the ecological implications of these results should be considered in light of the limitations inherent to laboratory studies. Therefore, conducting more laboratory and in-field studies, considering these factors, would be valuable in establishing the role of *A. araucaria* volatile compounds as semiochemicals for weevils. Beyond this, understanding the interactions between *A. araucana* volatiles and weevils is helpful in monitoring the forest situation through the quantity of these semiochemicals released by trees, or by developing devices that can be used in the field. Nowadays, mass trapping systems for insect pests are still widely used to control curculionids in coniferous forests [75], where single semiochemicals can be used as trap baits in traps or as part of blends. These types of studies and technologies could be useful to prevent further damage or alleviate the stress caused by insect pests.

## Figures and Tables

**Table 1:** Chemical composition of the volatile blends emitted from healthy and unhealthy *A. araucana* branches.

RT	KI_exp_	KI_lit_	Compound	Concentration (ng g^−1^)	
				Healthy	Unhealthy
9.27	922	914	Tricyclene	11.2 ± 4.8	8.2 ± 7.4
9.63	935	938	*α*-Pinene	47.3 ± 21.2	68.3 ± 61.6
10.01	948	953	Camphene	2.4 ± 1.0	-
10.9	976	976	Sabinene	2.7 ± 2.6	-
10.91	976	980	*β*-Pinene	2.7 ± 3.4	7.4 ± 11.6
12.08	1038	1013	Myrcene	368.3 ± 632.5	-
13.82	1072	1033	Limonene	4.0 ± 5.6	3.6 ± 3.1
15.79	1138	1143	Camphor	21.4 ± 17.0	61.2 ± 19.3
21.67	1349	1339	*δ*-Elemene	24.0 ± 41.5	5.2 ± 9.0
22.64	1385	1377	*α*-Copaene	29.0 ± 38.2	50.5 ± 31.5
23.02	1399	1392	*β*-Elemene	24.0 ± 41.5	5.3 ± 9.1
23.30	1411	1408	7-*epi*-Sesquithujene	-	5.0 ± 8.4
23.70	1428	1419	Caryophyllene	65.1 ± 21.6	64.6 ± 40.4
23.99	1440	1435	*γ*-Elemene	25.3 ± 43.8	-
24.02	1441	1438	*α*-Bergamotene	8.9 ± 14.2	24.4 ± 29.4
24.46	1459	1455	Humulene	52.3 ± 18.0	44.0 ± 30.8
24.51	1460	1459	Sesquisabinene	-	5.1 ± 8.9
25.05	1482	1492	Epicubebol	-	5.2 ± 9.0
25.15	1486	1446	*cis*-*β*-Farnesene	3.6 ± 6.3	15.8 ± 15.8
25.19	1487	1432	*β*-Copaene	24.2 ± 41.9	-
25.26	1490	1480	Germacrene D	5.2 ± 9.1	10.6 ± 18.4
26.00	1521	1513	*γ*-Cadinene	24.2 ± 41.9	20.6 ± 23.2
26.22	1531	1529	*β*-Cadinene	-	28.7 ± 25.2
26.41	1539	1509	*β*-Bisabolene	49.9 ± 22.9	47.8 ± 43.3
34.93	1938	1933	Hibaene	82.3 ± 57.8	140.7 ± 77.2
35.67	1977	-	Verticiol	-	17.4 ± 16.6
36.03	1995	1989	Trachylobane	46.4 ± 22.8	54.9 ± 21.1
36.44	2018	2015	Manoyl oxide	27.8 ± 39.0	35.9 ± 38.7
36.85	2041	-	Atiserene	18.3 ± 31.7	31.3 ± 40.4
36.94	2046	-	Kaurene	52.4 ± 19.0	37.4 ± 35.3
	Total amount			1022.9	799.1

RT: retention time (min); KI_exp_: calculated Kovats retention index; KI_lit_: Kovats retention index from the literature.

## Data Availability

The original contributions presented in this study are included in the article. Further inquiries can be directed to the corresponding author.
